# Prevalence and factors associated with neonatal mortality among neonates hospitalized at the National Hospital Nouakchott, Mauritania

**DOI:** 10.11604/pamj.2019.34.152.14683

**Published:** 2019-11-18

**Authors:** Abdellahi Weddih, Mohamed Lemine Cheikh Brahim Ahmed, Mariem Sidatt, Nessiba Abdelghader, Fatimatou Abdelghader, Abdi Ahmed, Saad bouh Regad, Khatry Makhalla, Jorg Heukelbach, Amina Barkat

**Affiliations:** 1 Ministry of Health, Pediatric Department, Nouakchott, Mauritania; 2 University of Nouakchott Al-Asriya, Nouakchott, Mauritania; 3 The Mauritanian Association for Scientific Research Development (AMDRS) and Researches Centre (MAALIM), Nouakchott, Mauritania; 4 Department of Community Health, School of Medicine, Federal University of Ceará , Fortaleza CE 60430-140, Brazil; 5 University Mohammed V, Rabat, Morocco

**Keywords:** Neonatal mortality, risk factors, hospitalization, neonates

## Abstract

**Introduction:**

Neonatal mortality remains a significant public health burden worldwide, with about 4 million deaths per year. To provide evidence for the implementation of prevention measures aimed at the reduction of neonatal mortality, we performed a study on factors associated with neonatal mortality at the Referral Hospital in Nouakchott, Mauritania.

**Methods:**

We conducted a cross-sectional study between January 2013 and December 2013 and included neonatal patients hospitalized at the National Referral Hospital (NRH). Data were collected by reviewing the medical charts and through questionnaires administered to the parents.

**Results:**

Two hundred and thirty-two (34.7%) of the 669 neonates included in the study died; 159 (71.3%) of deaths occurred during the first six days of life. Most neonates that died were born outside the hospital and admitted to NRH after birth (71.7%; 142/198). About 1/3 were transferred from other parts of the country outside of Nouakchott. Thirty (13.4%) of deaths were neonates born from teenage mothers. In bivariate analysis teenage mothers (RR=1.54; 95% CI: 1.15-2.05; p = 0.004), illiteracy of father (1.61; 1.13-23.0; p = 0.007), birth outside NRH (1.65; 1.28-2.13; p < 0.0001), low gestational age (3.28; 2.40-5.50; p < 0.0001), and low body temperature at admission (1.42; 1.11-1.83; p < 0.004) were significantly associated with neonatal death. In logistic regression analysis, low birth weight (adjusted odds ratio = 3.91; 95% confidence interval 1.69-9.05; p = 0.001), hypothermia (2.40; 1.12-5.14; p = 0.025), and birth outside the NRH (2.13; 1.02-4.45; p = 0.044) were independently associated with neonatal deaths.

**Conclusion:**

Neonatal mortality remains a significant burden in Mauritania. We identified different socioeconomic and clinical risk factors indicating the need for more intensified prenatal care and improved transport of high risk neonates, especially in the regions outside the capital.

## Introduction

**Figure 1:**
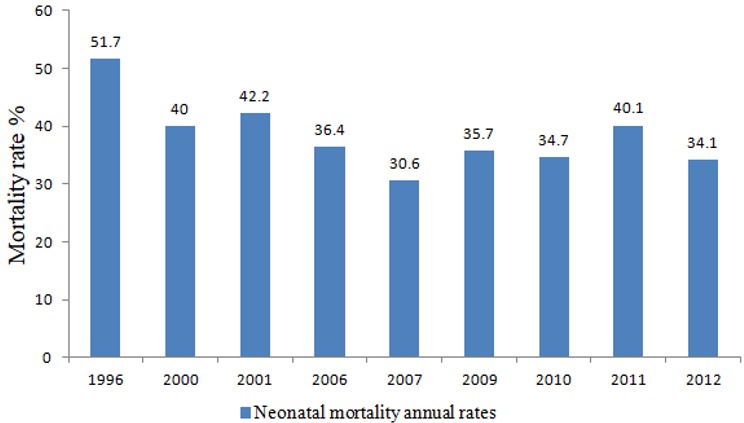
The annual changes of neonatal deaths in Nouakchott Mauritania

Neonatal deaths (defined as deaths among live births in the first 28 days of life) account for about 44% of deaths among children <5 years worldwide. In 2013, a total of about 2.8 million neonatal deaths have been estimated [1-3]. Of these, 99% occurred in developing countries, with the highest rates in sub-Saharan Africa (35 deaths per 1,000 live births in 2013) [1, 4]. The targets set by the Lancet Commission on Investing in Health and the Sustainable Development Goals (SDGs) for decreasing neonatal deaths is defined as 12 neonatal deaths per 1,000 livebirths by 2030 [4]. To meet this target, systematic practical interventions need to be implemented that are effective and efficient. Mauritania has a population of about 4 million, with 17% under the age of five years. Of these, about 22% are < one month of age [5, 6]. According to the United Nations Inter-agency Group for Child Mortality Estimation reports, neonatal mortality rates in Mauritania practically have remained unchanged during the last 30 years, and ranged between 41 per 1,000 live births in 1990 and 37 per 1,000 live births in 2010 [1, 3]. In addition, the annual changes of neonatal deaths at the National Referral Hospital (NRH) in Nouakchott, Mauritania was similar with these estimation reports (Figure 1). In 2013, the World Health Organization (WHO) estimated the neonatal mortality rate to be 34.8 per 1000 live births, with the most important single causes of death prematurity (37%), birth asphyxia & birth trauma (24%), and sepsis and other infectious conditions (21%) [3, 4]. To provide evidence for the implementation of specific prevention measures aimed at the reduction of neonatal mortality, we performed a cross-sectional study on factors associated with neonatal mortality at the Referral Hospital in Nouakchott, Mauritania.

## Methods

**Setting:** Mauritania is a vast country of about 1,030,700 km^2^ in size, but with a population less than 4 million. It is located between West and North Africa, surrounded by Senegal, Mali, Algeria and Western Sahara. The capital Nouakchott with about 1 million inhabitants corresponds to about one-fourth of the population of the country [6]. The National Referral Hospital (NRH) is the main pediatric hospital in Mauritania, located in Nouakchott. It is the largest pediatric service in the country with a bed capacity of 83. The pediatric services at the NRH receive high-risk neonates from different parts of the country. Neonates admitted and hospitalized at this hospital are a high risk group.

**Study population and design:** we performed a cross-sectional study. The target population consisted of neonates hospitalized for any reasons at the NRH in Nouakchott from 1^st^ January to 31^st^ December 2013.

**Data collection and analysis:** data were collected by reviewing the medical charts and through a structured questionnaire administered to the parents. The data collected contained the following variables: clinical diagnosis at admission, sex, maternal and neonate’s age, socioeconomic status and level of education of parents, level of education of mother, gestational age, number of antenatal care visits (0 and 1-3 or > 3), birth weight, place of birth (NRH or other), neonate’s body temperature at admission, mode of delivery, and outcome (survival/death). For classification of clinical diagnoses (congenital malformation, prematurity, birth asphyxia, infection), we adopted the International Classification of Diseases in its tenth revision (International Statistical Classification of Diseases and Related Health Problems ICD-10-WHO, version 2015). For bivariate and multivariate analyses assessing risk factors, survival of the neonate (survived or died within neonate period of 28 days) was defined as the binary outcome. Statistical significance of the differences between groups was assessed by applying chi squared and Fisher’s exact tests. We calculated adjusted Odds Ratios (aOR) with their 95% Confidence Intervals (CI) using logistic regression analysis. We included all variables with p < 0.05 from bivariate analyses in the multivariable model. Data were analyzed using SPSS version 22.0.

**Ethics consideration:** ethical clearance was obtained from the University of Nouakchott, Faculty of Medicine Ethical Review Board and the NRH administration. After parental consent, the additional data not included in the medical charts were collected from the neonates’ parents via questionnaires.

## Results

The study population consisted of 669 neonates hospitalized during the study period. Of these, 56.4% (376/667) were males, and almost ¾ had low birth weight (72.5%; 479/661). About ¼ of parents never had attended school, and the majority of mothers were housewives. A total of 232/669 (34.7%) died (Table 1). There was a high burden of early neonatal mortality with 159/223 (71.3%) of deaths occurring during the first six days of life, and 112/669 (17.0%) were pre-term births (<32 weeks). More than 13% of deaths were neonates born from teenage mothers (13.4%; 30/224). About one in six mothers never had visited any prenatal care or antenatal check-ups (15.7%; 34/216). Most neonates that died were born outside the hospital and admitted to NRH after birth (71.7%; 142/198). About 1/3 were transferred from other parts of the country outside of Nouakchott (Table 1). In bivariate analysis teenage mothers, illiteracy of father, birth outside NRH, low gestational age, and low body temperature at admission were significantly associated with neonatal death (Table 1). In multivariate analysis, low birth weight, hypothermia and birth outside NRH were independently associated with neonatal deaths (Table 2). Neonates with low birth weight had almost a fourfold chance of dying, as compared to norm weight neonates (Table 2).

**Table 1:** Bivariate analysis of socio-demographic and clinical factors associated with neonatal deaths at the Nouakchott Referral Hospital

Variable	Neonatal deaths N*	Neonatal deaths% (95% CI)	RR (95% CI)	P value
**Age**				
0-6 days	159/364	43.7% (38.4%-48.9%)	1.48 (1.16-1.87)	**<0.001**
7-28 days	64/217	29.5% (23.5%-35.9%)	Ref.	
**Sex:**				
Male	139/376	36.9% (32.1%-42.0%)	1.15 (0.93-1.43)	<0.08
Female	93/291	31.9% (26.8%-37.8%)	Ref.	
**Mother’s age:**				
<19 years	30/60	50.0% (36.6%-63.3%)	1.54 (1.15-2.05)	**0.004**
19-35 years	145/447	32.4% (28.1%-36.9%)	Ref.	
>35 years	49/131	37.4% (29.0%-46.5%)	1.15 (0.89-1.49)	0.14
**Level of education of mother:**				
Illiterate	29/80	36.2% (26.2%-47.5%)	0.99 (0.70-1.40)	0.48
Primary School or Quranic School	74/203	36.4% (30.0%-43.3%)	Ref.	
**Level of education of father:**				
Illiterate	26/53	49.0% (30.1%-58.4%)	1.61 (1.13-2.30)	**0.007**
Primary School or Quranic School	53/175	30.2% (23.4%-37.7%)	Ref.	
**Socioeconomic status of parents:**				
Low	95/352	26.9% (22.4%-31.8)	0.49 (0.39-0.61)	**<0.0001**
Medium or high	83/151	54.9% (46.3%-62.9%)	Ref.	
**Body temperature at admission:**				
Hypothermia (<36°C)	89/181	49.1% (41.4%-56.9%)	1.42 (1.11-1.83)	**<0.004**
Normothermia (36°C-37.5°C)	63/183	34.4% (27.3%-41.5%)	Ref.	
Hyperthermia (>37.5°C)	30/105	28.5% (20.0%-38.0%)	0.82 (0.57-1.19)	0.30
**Place of birth:**				
Outside NRH	142/319	44.5% (38.8%-50.1%)	1.65 (1.28-2.13)	**<0.0001**
NRH	56/208	26.9% (21.1%-33.6%)	Ref.	
**Birth weight:**				
Low	157/479	32.7% (28.6%-37.1%)	0.59 (0.48-0.72)	**<0.0001**
Normal	70/126	55.5% (46.8%-64.2%)	Ref.	
Overweight	4/56	7.1% (1.7%-17.8%)	0.12 (0.04-0.33)	**<0.0001**
**Crying at birth:**				
Late	66/212	31.1% (25.0%-37.7%)	1.00 (0.74-1.33)	0.9
Immediately	60/193	31.0% (24.8%-38.3%)	Ref.	
**Gestational age:**				
<32 weeks	68/112	60.7% (50.8%-69.6%)	3.28 (2.40-4.50)	**<0.0001**
32-37 weeks	41/222	18.4% (13.5%-24.3%)	Ref	
>37 weeks	116/317	36.6% (31.2%-42.3%)	1.98 (1.45-2.70)	**<0.0001**
**No. of antenatal visits:**				
0	34/149	22.8% (16.1%-30.2%)	0.36 (0.26-0.50)	**<0.0001**
1-3	69/141	48.9% (40.4%-57.4%)	0.78 (0.63-0.96)	**<0.001**
>3	113/181	62.4% (54.6%-69.6%)	Ref.	
**Mode of delivery:**				
Cesarean sction	48/133	36.1% (27.8%-45.1%)	1.06 (0.82-1.37)	0.32
Normal	179/527	33.9% (29.9%-38.1%)	Ref.	
**Total**	**232/669**	**34.7% (31.0%-38.4%)**		

* Data not available in all cases

**Table 2:** Multivariate analysis of factors independently associated with neonatal deaths

Variable	Adjusted odds ratio	95% CI	P value
Low birth weight	3.91	1.69-9.05	0.001
Hypothermia	2.40	1.12-5.14	0.025
Born outside NRH	2.13	1.02-4.45	0.044

## Discussion

This is the first systematic study providing information about neonatal mortality among neonates hospitalized in Mauritania. The extremely high neonatal mortality with more than 1/3 of neonates dying is similar to 35.5% recorded by Debelew *et al.* (2014) from Ethiopia [7]. The study indicates that neonatal mortality was largely associated with socioeconomic status, and clinical features, such as low birth weight, hypothermia, transfer condition, and low gestational age. In general, neonatal mortality was lower than the national estimate (39%) [3], but much higher than the findings of recent similar studies from other African countries [8-11]. The high percentage of neonatal deaths could be explained by the absence of adequate health services leading to insufficient prenatal care, and late presentation at NRH, and the low socioeconomic conditions of a considerable part of Mauritania’s population. We have previously shown that many of the neonates who transferred to this hospital have severe complications with a high risk of death. In fact, hypothermia and transfer condition, which can be seen as indicators for late presentation at health services were identified as risk factors. Similar results have been described from other developing countries [1, 8]. Others have also reported a high proportion of neonatal mortality to occur among neonates outborn and transferred for hospitalization [8, 12]. A higher proportion of neonatal deaths was reported among gender males up to 59.9%, this predominance was also found in many other studies [13-15]. However, in our study, there was no significant difference between male and female in mortality. The majority of neonatal deaths occurred during the first week of life; this finding is similar to 65% recorded by Welaga *et al.* (2013) from Ghana [16]. Authors from other countries found even higher rates [8, 10, 17]. These results show that this period is the most critical period for monitoring the quality of service and the interventions should be intensified to reduce the neonatal mortality. We found that gestational abnormalities and perinatal specified infections were the major causes of deaths within the first week. Similarly, other studies reported prematurity to be the leading cause of death during the first week of life [2, 4, 8].

Neonatal mortality was highest in neonates from teenage mothers. In fact, teenage pregnancies have to be regarded as a high risk situation. Consequently, other authors described previously that a high proportion of neonatal mortality occurred among young mothers [12, 18]. Father’s education level was significantly associated with neonatal mortality, but not mother’s education level. In Mauritanian society, fathers are the heads of the families, and father’s education level may thus play in contrast to the mother’s education level the principal role, as their socioeconomic status and education largely defines the general family situation, and also defines health care seeking behavior during critical conditions. Yaya *et al*. (2014) also reported that parental education has a positive correlation with better health of children [19]. In Nepal, having a partner with no formal education was significantly associated with increased neonatal mortality [9]. Number of antenatal care visits was significantly associated with neonatal deaths; more than 3 antenatal care visits were associated with a higher number of deaths. This result is remarkable and different from the finding of other studies that have been suggested that attending the recommended four antenatal care visits could reduce neonatal mortality [20-22]. Our finding may be explained by the reality that health education is weak at public health facility centers in Mauritania. We also found a highest risk of deaths among neonates that were born with low birth weight. Authors from other countries had found similar results [4, 8, 12, 23, 24]. Our study is subject to limitations, especially missing data. There was an absence of the maternal file of certain neonates, transferred from other sites or inborn at the hospital. We had lost to follow up some cases because of the time pressure on staff. In addition, there is a major lack of similar studies from Mauritania, and data can only be compared to studies conducted in other countries.

## Conclusion

Our study evidenced an extremely high burden of neonatal mortality at a major hospital center in Mauritania, and identified risk factors indicating the need for more intensified prenatal care and improved transport of high risk neonates, especially in the regions outside the capital. There is a need to conduct further studies in order to better describe the epidemiology of neonatal mortality, and to identify the most effective and efficient national intervention measures.

### What is known about this topic

- Timely and appropriate interventions in the first 28 days of life reduce neonatal mortality;
- Based on WHO estimation, Mauritania is one of the countries with highest neonatal mortality in Africa, but systematic data are lacking.

### What this study adds

- Neonatal mortality among neonates hospitalized (34.7%) is very high at the National Referral Hospital in Mauritania;
- Main risk factors for neonatal mortality include illiteracy, teenage pregnancy and birth outside the Referral Hospital, low gestational age, low birthweight, and hypothermia.

## Competing interests

The authors declare no competing interests.
